# Rescue of PFOS-induced human Sertoli cell injury by overexpressing a p-FAK-Y407E phosphomimetic mutant

**DOI:** 10.1038/s41598-017-15671-4

**Published:** 2017-11-17

**Authors:** Haiqi Chen, Ying Gao, Dolores D. Mruk, Xiang Xiao, Constance M. John, Paul J. Turek, Wing-yee Lui, Will M. Lee, Bruno Silvestrini, C. Yan Cheng

**Affiliations:** 1 The Mary M. Wohlford Laboratory for Male Contraceptive Research, Center for Biomedical Research, Population Council, 1230 York Ave, New York, New York, 10065 USA; 2 Department of Reproductive Physiology, Zhejiang Academy of Medical Sciences, Hangzhou, Zhejiang, 310013 China; 3 MandalMed, San Francisco, CA 94107 USA; 4 The Turek Clinic, San Francisco, CA 94133 USA; 5 School of Biological Sciences, University of Hong Kong, Hong Kong, China; 6 SBM Srl Pharmaceuticals, Rome, 00167 Italy

## Abstract

PFOS induces Sertoli cell injury using testicular cells isolated from rodent testes, but it remains unknown if PFOS has similar effects in humans. Herein, we maintained human Sertoli cells in a mitotically active state *in vitro*, thus enabling transfection experiments that altered gene expression to explore the molecular mechanism(s) underlying toxicant-induced cell injury. Human Sertoli cells obtained from men at ages 15, 23, 36 and 40 were cultured *in vitro*. These differentiated Sertoli cells remained mitotically active when cultured in the presence of 10% FBS (fetal bovine serum), with a replication time of ~1–3 weeks. At ~80% confluency, they were used for studies including toxicant exposure, immunoblotting, immunofluorescence analysis, tight junction (TJ)-permeability assessment, and overexpression of BTB (blood-testis barrier) regulatory genes such as FAK and its phosphomimetic mutants. PFOS was found to induce Sertoli cell injury through disruptive effects on actin microfilaments and microtubule (MT) organization across the cell cytosol. As a consequence, these cytoskeletal networks failed to support cell adhesion at the BTB. Overexpression of a FAK phosphomimetic and constitutively active mutant p-FAK-Y407E in these cells was capable of rescuing the PFOS-induced injury through corrective cellular organization of cytoskeletal elements. Summary: PFOS induces human Sertoli cell injury which can be rescued by overexpressing p-FAK-Y407E mutant.

## Introduction

Perfluorooctanesulfonate (or perfluorooctanesulfonic acid) (PFOS) and its related products (e.g., perfluorooctanoic acid (PFOA)) were first produced by 3M Company in 1949. They have been widely used in consumer products, as stain repellents and act by reducing the surface tension of water, serving as fabric and clothing protectors^1^. PFOS use has since expanded and it can now be found in many consumer products such as textiles, leather, and general cleaning products. But toxic effects of PFOS as an endocrine disrupter have also been realized, first in wildlife and then in humans and include developmental defects, cancer risk, and neonatal mortality^2–5^. As such, in 2000, 3M phased out its production in the U.S.^6^. In 2006, the European Union banned the use of PFOS, followed by Canada in 2009. To date, PFOS is still used as a stain repellent in consumer products in China, with an estimated ~200 tons produced annually^7,8^. Human exposure to PFOS is low, with an acceptable PFOS daily intake (ADI)/tolerable daily in-take (TDI) dose determined to be 150 ng/kg b.w. per day or ~12 µg/per man per day^9^. However, the half-life of PFOS and PFOA are relatively long, at 5.4 years and 3.5 years, respectively^9–11^. Thus, a considerable amount of PFOS or PFOA can accrue in organs over time. In fact, blood levels of PFOS in humans are on the rise, increasing from 29.5 ng/mL in 1974 to 34.7 ng/mL in 1989^12^. Given the potential for PFOS and its substrates to act as endocrine disruptors, we sought to evaluate the effect of PFOS on human Sertoli cells *in vitro*.

PFOS and its related compounds are peroxisome proliferators and agonists of peroxisome proliferator-activated receptors^2^. Since peroxisome proliferator-activated receptors are nuclear receptors and transcription factors that are known to regulate cellular differentiation, metabolism and development^13,14^, it is not unexpected that PFOS/PFOA might induce developmental defects in wildlife and humans^2–5^. However, what is still unclear is the molecular mechanism by which PFOS (at ~20–40 µM) might induce testis injury, besides simply acting as an endocrine disruptor^9^. In rats, PFOS has been shown to induce Sertoli cell injury in primary Sertoli cell cultures by blocking Sertoli cell gap junction (GJ) communication through disruptive changes in the organization of actin microfilaments across the Sertoli cell cytosol, and likely involves the p-FAK-Tyr^407^ signaling protein^9^. In humans, it is unknown whether PFOS can induce Sertoli cell injury. Due to the ongoing use of PFOS in consumable products in particular in China, we thought it pertinent to investigate: (i) if PFOS would induce human Sertoli cell injury, and (ii) if p-FAK-Tyr^407^, a signaling protein known to promote rat Sertoli cell function by promoting the integrity of the Sertoli cell blood-testis barrier (BTB), would rescue PFOS-induced disruption and Sertoli cell injury in human Sertoli cells cultured *in vitro* ^15,16^. This knowledge would provide much needed insight into PFOS-mediated testis injury and inform us further on potential mechanisms of environmental toxicant-induced male reproductive dysfunction.

## Results

### PFOS perturbs the human Sertoli cell TJ-permeability barrier through alterations in steady-state levels, and localization, of BTB-associated proteins

**Figure 1:**
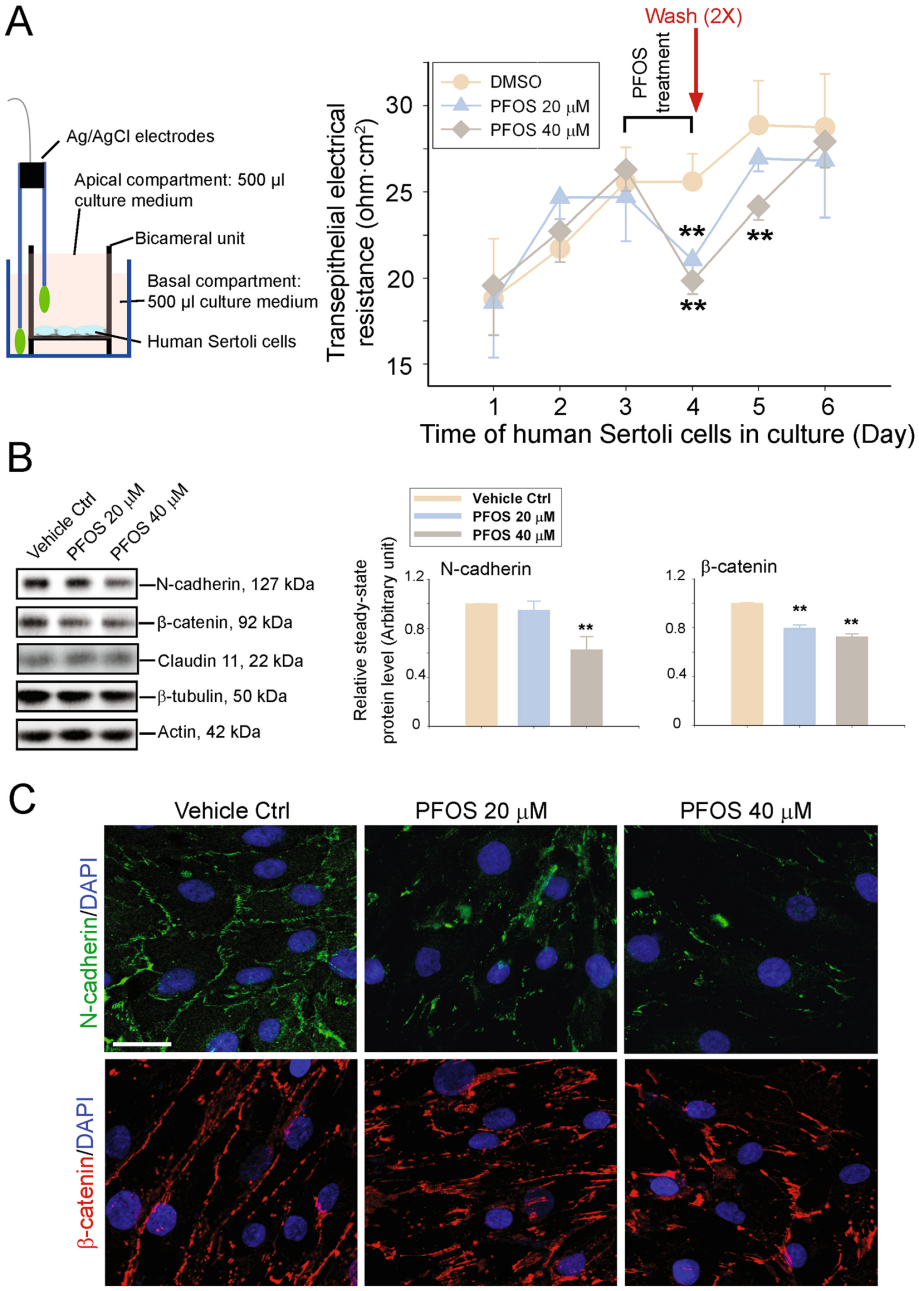
Effects of perfluorooctanesulfonate (PFOS) on the expression and localization of BTB-associated cell adhesion proteins in cultured human Sertoli cells. (**A**) On the left panel, a schematic drawing that illustrates the use of a Millipore Millicell-ERS-2 unit and the attached electrode pair to monitor the human Sertoli cell TJ-permeability barrier function when cells were plated on fibronectin-coated bicameral units. On the right panel, typical results of a TER experiment that monitored the effects of PFOS (20 and 40 µM) on the human Sertoli cell TJ-permeability barrier function *in vitro*. PFOS treatment was performed on day 3 and incubated with cells for 24 h, thereafter, TER reading was taken and PFOS was removed by rinsing cells twice with the culture medium, and then replenished with fresh medium for incubation. Each data point is a mean ± SD of quadruplicate bicameral units from an experiment of *n* = 4 independent experiments which yielded similar results. **P < 0.01 by Student’s *t*-test compared between PFOS-treated and vehicle control (DMSO) cells. (**B**) Cultured human Sertoli cells at ~70–80% confluency were exposed to PFOS (20 and 40 μM) for 24 hr. Thereafter, cells were harvested for lysate preparation and for immunoblotting (~30 µg per lane) (left panel). The histogram in the right panel summarizes results of immunoblottings as shown in the left panel. Protein bands were densitometrically scanned, and normalized against β-actin with the vehicle control arbitrarily set at 1 for statistical comparison. Each bar is a mean ± SD of *n* = 3 independent experiments. **P < 0.01 by Student’s *t*-test compared between PFOS-treated and vehicle control (DMSO) cells. Uncropped images of immunoblots can be found in Figure S1. (**C**) Immunofluorescence analysis of BTB-associated proteins N-cadherin (green fluorescence) and β-catenin (red fluorescence). Cell nuclei were visualized by 4′,6-diamidino-2-phenylindole (DAPI). Scale bar, 40 μm, which applies to other micrographs.

When human Sertoli cells were plated at ~90% confluency on human fibronectin-coated bicameral units and cultured in DMEM/F12 medium with other additives and antibiotics in 24-well dishes (Fig. 1A, left panel), they were capable of establishing a TJ-permeability barrier which could be monitored by quantifying TER across the cell epithelium as noted in Fig. 1A (right panel). Furthermore, PFOS showed a dose-dependent perturbation in the Sertoli cell TJ-permeability barrier (Fig. 1A). These disruptive effects of PFOS on the TJ barrier were likely mediated by down-regulation and redistribution of the BTB-associated proteins, such as N-cadherin (an integrated membrane basal ES protein) and its adaptor ß-catenin (Fig. 1B,C; Figure S1). For instance, N-cadherin no longer localized neatly at the human Sertoli cell-cell interface, but was rapidly internalized, possibly undergoing endocytic vesicle-mediated degradation (Fig. 1C), thereby impeding its expression as noted in Fig. 1B.

### PFOS perturbs human Sertoli cell-TJ permeability barrier through its disruptive effects on the organization of actin- and MT-based cytoskeletons

**Figure 2:**
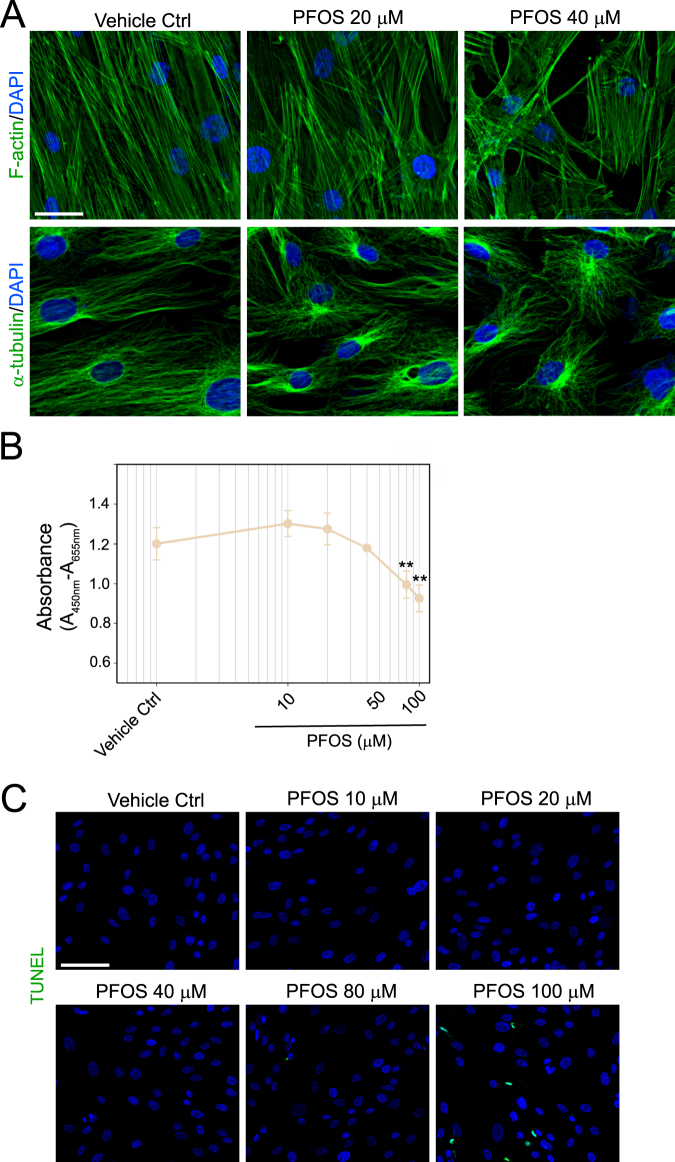
PFOS (at 20 and 40 µM) perturbs the organization of actin- and microtubule (MT)-based cytoskeletons in human Sertoli cells without inducing cytotoxicity and apoptosis. (**A**) Human Sertoli cells at 70–80% confluency were treated with PFOS for 24 hr. Immunofluorescence analysis of human Sertoli cell F-actin (green fluorescence) and α-tubulin (green fluorescence, the building blocks of MTs) network was then performed. Cell nuclei were visualized by DAPI. Scale bar, 40 μm. PFOS induced defragmentation of actin microfilaments in which actin microfilaments no longer stretched across the Sertoli cell noted in control cells. PFOS also induced retraction of MTs from cell cytosol whereas MTs stretched across the entire cell cytosol in control cells. (**B**) Human Sertoli cells were exposed to PFOS at 10, 20, 40, 80 and 100 μM *vs*. vehicle control for 24 hr, and cell cytotoxicity was monitored by tetrazolium dye XTT-based assay. Each data point is a mean ± SD of triplicate wells from an experiment with *n* = 3 independent experiments and yielded similar results. **P < 0.01, 1-way ANOVA. (**C**) Human Sertoli cells were exposed to PFOS at 10, 20, 40, 80 and 100 μM *vs*. vehicle control for 24 hr, and were subjected to an apoptosis assay in which apoptotic cells displayed green fluorescence in the nuclei. Cell nuclei were visualized by DAPI. Scale bar, 120 μm.

We sought to examine the underlying mechanism by which PFOS induced human Sertoli cell TJ-barrier disruption by monitoring the organization of actin- and MT-based cytoskeletons (Fig. 2A). Indeed, treatment of human Sertoli cells with PFOS perturbed F-actin organization and induced extensive truncation of actin microfilaments across the Sertoli cell cytosol. Interestingly, actin microfilaments in cells exposed to PFOS no longer stretched across the entire cytosol unlike controls (Fig. 2A). Microtubules (MTs) visualized by α-tubulin (one of the two building blocks of MTs) were no longer stretched across the entire cell cytosol, but showed considerable dose dependent retraction, keeping close to the nuclei following PFOS treatment (Fig. 2A). At doses up to 40 µM, PFOS had no cytotoxic effects on human Sertoli cells when assessed by the XTT assay (Fig. 2B) nor did it induce unwanted cell apoptosis based on TUNEL assay (Fig. 2C).

### PFOS perturbs F-actin organization in human Sertoli cells through changes in the spatial expression of actin regulatory proteins Eps8 and Arp3

**Figure 3:**
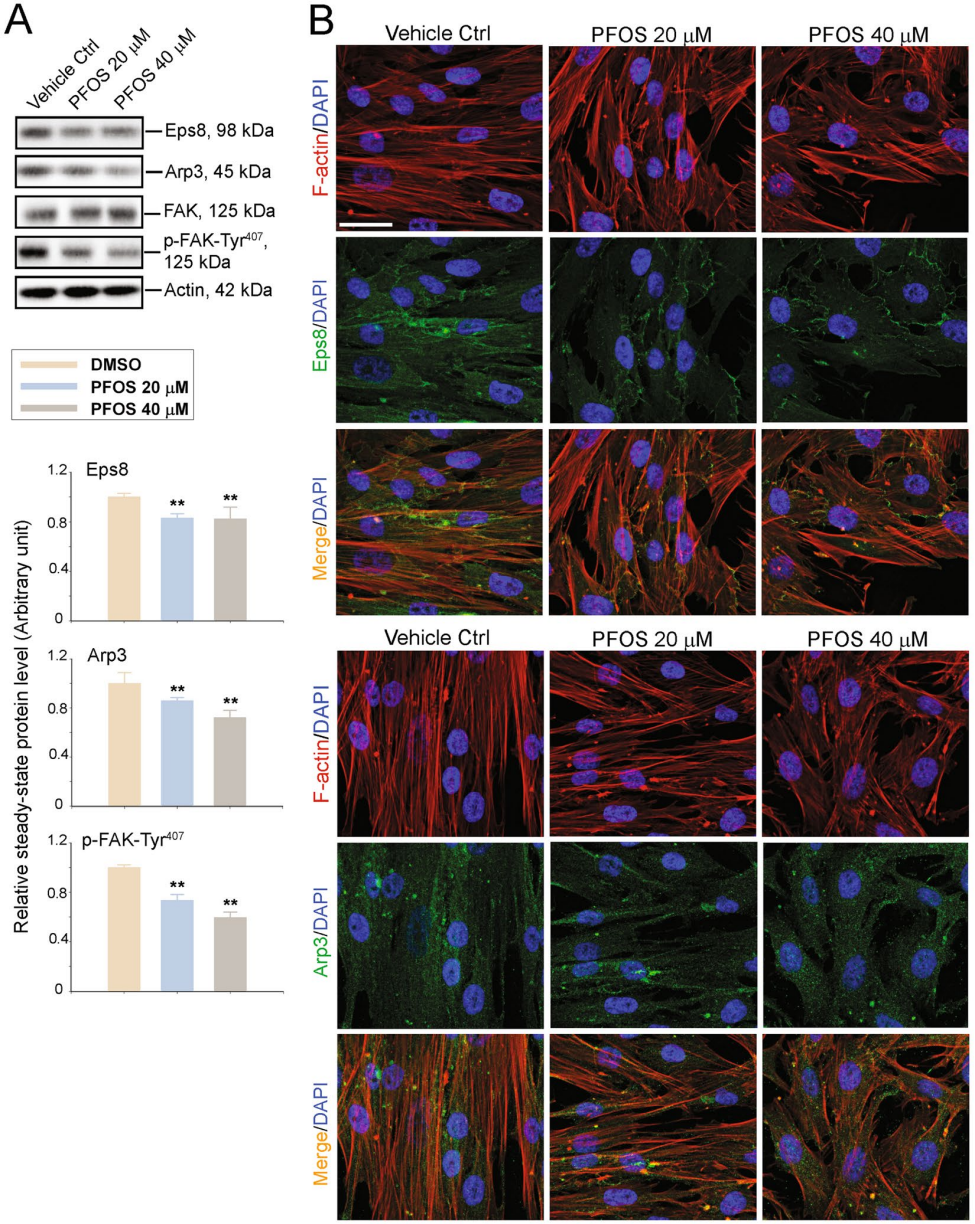
PFOS-induced disruption of the actin-based cytoskeleton in human Sertoli cells is mediated via changes in the spatial expression of actin binding/regulatory proteins. (**A**) The steady-state protein levels of actin regulatory proteins Eps8 (an actin barbed end capping and bundling protein known to maintain actin microfilaments into a bundled network to support ES function), and Apr3 (a branched actin polymerization protein known to convert bundled actin filaments into a branched/unbundled network to facilitate ES disruption), as well as F-actin regulatory signaling protein FAK in particular p-FAK-Tyr^407^ in human Sertoli cells following PFOS treatment were assessed by immunoblottings. ß-Actin served as a protein loading control. These immunoblotting data were summarized in the histograms shown on the lower panel. Each bar is a mean ± SD of *n* = 3 independent experiments. Protein level in vehicle controls cells was arbitrarily set at 1 against which statistical comparison was performed. **P < 0.01 by Student’s *t* test compared between PFOS-treated and vehicle control (DMSO) cells. Uncropped images of immunoblots can be found in Figure S2. (**B**) Dual-labeled immunofluorescence analysis of F-actin (red fluorescence) *vs*. actin regulatory protein Arp3 (green fluorescence) or Eps8 (green fluorescence). Cell nuclei were visualized by DAPI. Scale bar, 40 μm, which applies to all other micrographs.

Studies in the rat testis have shown that Eps8, an actin barbed end capping and bundling protein^17,18^, supports ES (ectoplasmic specialization, a testis specific atypical adherens junction) function by conferring actin microfilaments into a bundled configuration^19^. On the other hand, Arp3, a component of the Arp2/3 complex which is a barbed end nucleation protein in the testis^20^, is known to confer branched actin polymerization, promoting actin filaments to assume a branched actin/unbundled configuration^18,21^. Interestingly, the kinetics of actin polymerization was also shown to be modulated by overexpression of p-FAK-Tyr^407^ and its phosphomimetic (i.e., constitutively active, such as p-FAK-Y407E) mutant or its non-phosphorylatable (i.e., constitutively inactive, such as p-FAK-Y407F) mutant^22^, supporting the notion that p-FAK-Tyr^407^ promotes Sertoli cell TJ-barrier function in the rat testis. In short, the differential functions of Eps8 and the Arp2/3 complex confer plasticity to the actin microfilaments at the ES, making this ultrastructure highly flexible by converting the actin microfilament network at the ES between a bundled and unbundled/branched configuration to support spermatid transport across the epithelium during the epithelial cycle in the rat testis. Using human Sertoli cell epithelium, PFOS was shown to be capable of inducing down-regulation of the expression of Eps8, Arp3 and also p-FAK-Tyr^407^ (Fig. 3A; Figure S2). More importantly, PFOS grossly perturbed the distribution of Eps8, such that it was no longer found along actin microfilaments across the human Sertoli cell, and no longer supporting the organization of linear actin filaments (Fig. 3B, upper panel). PFOS also perturbed the organization of Arp3 (Fig. 3B, lower panel). These changes led to the truncation of actin filaments across the human Sertoli cell following PFOS treatment, suggesting that PFOS exerts its disruptive effects on the organization of the F-actin network in human Sertoli cells through an alteration on the spatial expression and localization of these actin regulatory proteins.

### PFOS perturbs MT organization in human Sertoli cells through changes in the spatial expression of MT +TIP protein EB1

**Figure 4:**
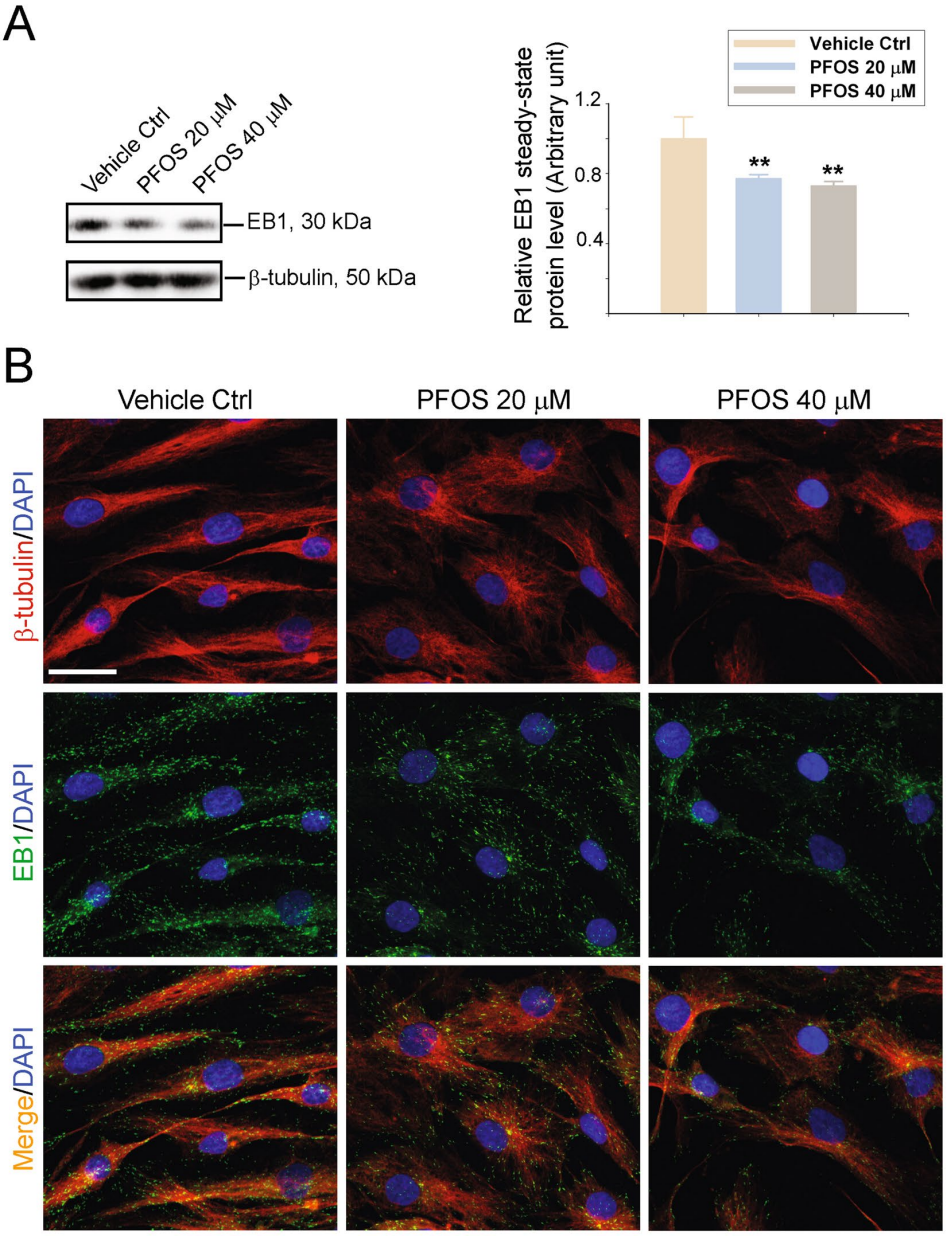
PFOS-induced disruption of the MT-based cytoskeleton in human Sertoli cells is mediated via changes in the spatial expression of microtubule +TIP protein EB1. The steady-state protein levels of EB1 (end binding 1, a microtubule plus (+) end tracking protein known to stabilize MT filaments) was considerably reduced in human Sertoli cells following PFOS treatment when assessed by immunoblottings. ß-Tubulin served as a protein loading control. Immunoblotting data were summarized in the histogram shown on the right panel. Each bar is a mean ± SD of *n* = 3 independent experiments. Protein level in vehicle controls cells was arbitrarily set at 1 against which statistical comparison was performed. **P < 0.01 by Student’s *t*-test compared between PFOS-treated and vehicle control (DMSO) cells. Uncropped images of immunoblots can be found in Figure S3. (**B**) Dual-labeled immunofluorescence analysis of ß-tubulin (red fluorescence, the building blocks of MTs) and EB1 (green fluorescence). Cell nuclei were visualized by DAPI. Scale bar, 40 μm, which applies to all other micrographs.

EB1 is a known +TIP protein, predominantly found at the (+)-end (i.e., fast growing end) of MT^23,24^. It has recently been shown to be an important MT regulator by stabilizing MTs at the ES in rat testes^25^. PFOS was found to down-regulate the expression of EB1 in human Sertoli cells (Fig. 4A; Figure S3). More important, PFOS also perturbed the distribution of EB1 across the Sertoli cell epithelium. Instead of stretching across the entire Sertoli cell cytosol to support MT organization, EB1 was found to retract from the cell periphery and stay localized to the cell nuclei (Fig. 4B). These changes caused MTs to retract from across the cell cytosol and to also localize more closely to the cell nuclei following PFOS treatment (Fig. 4B). These findings support the notion that PFOS disrupts MT organization in human Sertoli cell epithelium through its disruptive changes on the spatial expression and localization of MT binding protein EB1.

### p-FAK-Tyr^407^ promotes human Sertoli cell actin- and MT-based cytoskeletal organization to support BTB-associated protein distribution

**Figure 5:**
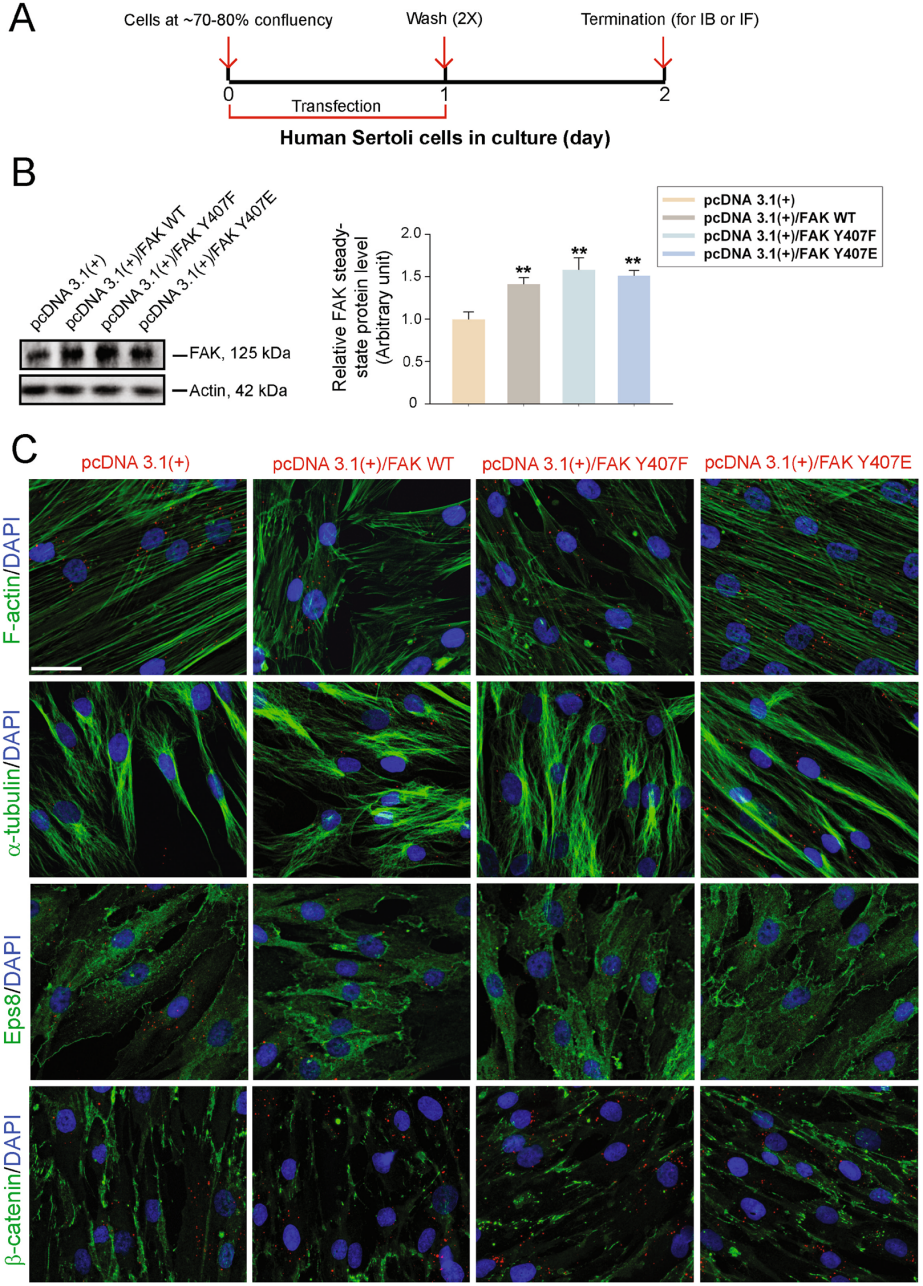
Effects of overexpression of the constitutively active phosphomimetic mutant of p-FAK-Y407E *vs*. the WT (wild-type), the constitutively inactive mutant of p-FAK-Y407F and control (empty vector, pcDNA 3.1(+)). (**A**) Regimen used for the experiment reported herein in which human Sertoli cells at ~70–80% confluency were transfected with corresponding plasmid DNA for 24 hr. Cells were allowed to recover for 24 hr before termination. (**B**) Overexpression of FAK using different mutants *vs*. the WT in human Sertoli cells was confirmed by immunoblotting (left panel). ß-Actin served as a protein loading control. These findings were summarized in the histogram shown on the right panel. Each bar is a mean ± SD of *n* = 3 independent experiments using different batches of Sertoli cells. **P < 0.01 by Student’s *t*-test. Uncropped images of immunoblots can be found in Figure S4. (**C**) Immunofluorescence analysis of F-actin (green fluorescence) and α-tubulin (green fluorescence), as well as F-actin regulatory protein Eps8 (green fluorescence) and adhesion protein β-catenin (green fluorescence) were shown. Successful transfection was confirmed by red fluorescence wherein plasmid DNA was labeled with Cy3 dye using a Mirus Label*IT* Tracker Intracellular Nucleic Acid Localization Kit. Cell nuclei were visualized by DAPI. Scale bar, 40 μm, which applies to all other micrographs.

Using the regimen outlined in Fig. 5A, overexpression of different mutants, namely the phosphomimetic (i.e., constitutively active) mutant p-FAK-Y407E, the non-phosphorylatable (i.e., constitutively inactive) mutant p-FAK-Y407F *vs*. FAK-WT and the empty vector alone (pcDNA3.1(+)) in human Sertoli cell epithelium was performed by transfecting human Sertoli cells with the corresponding plasmid DNA. This was found to induce an increase in the steady-state protein level of FAK by immunoblotting (Fig. 5B; Figure S4), even though this antibody failed to distinguish an increase in the expression of the corresponding p-FAK forms. Overexpression of p-FAK-Y407E was found to promote the organization of F-actin, MTs (noted by staining that visualized α-tubulin, the building block of MTs) and spatial localization of Eps8 and ß-catenin (Fig. 5C). Interestingly, overexpression of either the WT FAK or the p-FAK-Y407F mutant perturbed the organization of F-actin and MT, and the spatial distribution of Eps8 and ß-catenin in human Sertoli cells (Fig. 5C). Nonetheless, these findings are in agreement with earlier studies in rat Sertoli cell epithelium maintained *in vitro* that p-FAK-Y407E promotes Sertoli cell BTB integrity whereas p-FAK-Y407F induces Sertoli cell BTB disruption, through their effects to maintain or disrupt the organization of actin microfilaments across the rat Sertoli cell cytosol, respectively^22^. We next examined if the protective effects of p-FAK-Y407E on the Sertoli cell actin- and MT-based cytoskeletal function would rescue PFOS-mediated disruptive effects on cytoskeletal organization in human Sertoli cells.

### Rescue of PFOS-mediated disruption on actin- and MT-based cytoskeletal organization through overexpression of a p-FAK-Y407E mutant in human Sertoli cell epithelium

**Figure 6:**
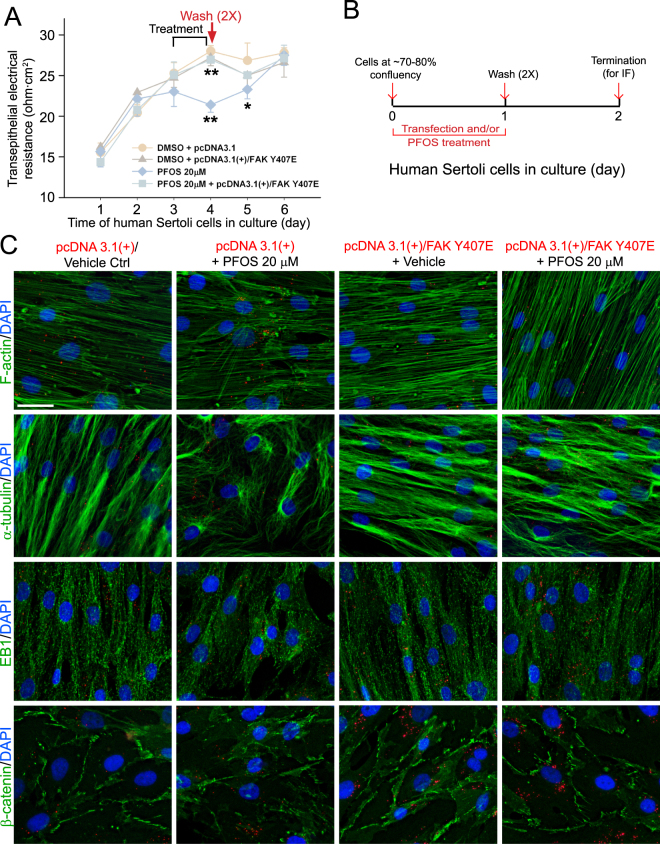
Overexpression of p-FAK-Y407E phosphomimetic mutant rescues human Sertoli cells from the disruptive effects of PFOS regarding their cytoskeletal and cell adhesion function. (**A**) Results of a typical TER experiment that monitored the protective effects of p-FAK-Y407E constitutively active phosphomimetic mutant on PFOS (20 µM)-mediated disruption of the human Sertoli cell-TJ permeability barrier function. Cells were transfected with the corresponding plasmid DNA and/or treated with PFOS in specified cell groups on day 3 for 24 hr. Thereafter, TER readings were obtained, and PFOS was then removed by rinsing cells twice with fresh medium (annotated by red arrow), and cells were replenished with fresh medium without PFOS to allow recovery of the TJ-barrier function. TER reading was obtained on a daily basis. Each data point is a mean ± SD of triplicate bicameral units from an experiment. A total of n = 3 independent experiments were performed using different batches of human Sertoli cells which yielded similar results. *P < 0.05; **P < 0.01 by Student’s t-test compared between PFOS-treated and vehicle control groups, or between PFOS-treated vs. PFOS+FAK-Y407E phosphomimetic mutant groups. (**B**) Regimen used for the experiment summarized in (**C**) in which human Sertoli cells at ~70–80% confluency were transfected with either empty vector, FAK-WT or FAK-Y407E plasmid DNA, and with or without PFOS for 24 hr. Cells were allowed to recover for 24 hr before termination and used for IF. (**C**) Immunofluorescence analysis of F-actin (green fluorescence) and β-tubulin (green fluorescence) network as well as microtubule regulatory protein EB1 (green fluorescence) and adhesion protein β-catenin (green fluorescence) in human Sertoli cells. Overexpression of p-FAK-Y407E, but not FAK-WT, in human Sertoli cells together with PFOS treatment was capable of rescuing these cells from the disruptive effects of PFOS regarding the organization of F-actin and MTs in these cells. FAK-Y407E overexpression also promoted proper spatial expression of EB1 across the Sertoli cell and proper localization of ß-catenin at the Sertoli cell-cell interface. Successful transfection was confirmed by red fluorescence wherein plasmid DNA was labeled with Cy3. Cell nuclei were visualized by DAPI. Scale bar, 40 μm, which applies to all other micrographs.

We next used a physiological assay to monitor if overexpression of a constitutively active phosphomimetic mutant FAK-Y407E could rescue the PFOS-induced Sertoli cell TJ-permeability disruption. Indeed, overexpression of FAK-Y407E mutant was effective to block the PFOS-induced Sertoli TJ-barrier disruption on day 4 (i.e., 24 hr after treatment with PFOS), making the TJ-barrier similar to the control (empty vector alone) cells but significantly different from the PFOS-treated cells (see PFOS+FAK Y407E *vs*. PFOS cell group) (Fig. 6A). By day 5, overexpression of FAK-Y407E also rendered PFOS treatment in the PFOS + FAK Y407E cells similar to control cells (i.e., overexpression of pcDNA3.1 empty vector alone) but the TJ-barrier in the PFOS-treated cells remained disrupted (Fig. 6A), illustrating the ability of FAK-Y407E to rescue cells from the damaging effects of PFOS. Using the regimen shown in Fig. 6B, involving overexpression of FAK-Y407E, the constitutively active phosphomimetic mutant, but not FAK WT, was also found to effectively rescue human Sertoli cells from the disruptive effects of PFOS on both actin- and MT-based cytoskeletons based on immunofluorescence analysis (Fig. 6C). The protective effects of FAK-Y407E appeared to be mediated by maintaining the spatial expression of EB1 that promoted MT organization even in the presence of PFOS (Fig. 6C). Due to the protective effects of FAK-Y407E on the F-actin network in Sertoli cell, the localization of BTB-associated adaptor protein ß-catenin was also maintained in the presence of PFOS (Fig. 6C). These findings illustrate that p-FAK-Tyr^407^, besides serving as a crucial signaling molecule that promotes Sertoli cell TJ-permeability barrier function in the rat testis^22^, is also an important signaling protein in the human Sertoli cell BTB. Collectively, these data also support earlier findings that PFOS exerts its damaging effects in the rat testis through disruptive changes in the organization of actin microfilaments in Sertoli cells via p-FAK-Tyr^407^ ^26^.

## Discussion

The observation that the actin- or the MT-based cytoskeleton in human Sertoli cells is the target of PFOS is consistent with earlier findings in rat studies in which PFOS induced defragmentation of actin microfilaments in primary Sertoli cell cultures, thereby perturbing the Sertoli cell TJ-permeability barrier function *in vitro* ^26^. Also, cadmium and bisphenol A are known to cause Sertoli cell injury in humans by inducing defragmentation of actin microfilaments across the human Sertoli cell cytosol, and interfering with the distribution of BTB-associated proteins such as ZO-1, N-cadherin and ß-catenin through changes in the spatial expression of Eps8 and Arp3^16^. However, it was unclear whether PFOS also exerted disruptive effects on Sertoli cells, in particular in humans, through changes in the organization of MTs besides actin microfilaments. In this context, it is of interest to note that MTs are a primary target of other toxicants such as 2,5-hexanedione and carbendazim^27–29^. For instance, colchicine and carbendazim are known to induce germ cell exfoliation by disrupting the rat Sertoli cell MT-cytoskeletal network^30–34^ wherein carbendazim (a fungicide) exerts its disruptive effects by inhibiting tubulin polymerization, thereby blocking MT assembly^35^. In all, these changes lead to failed spermatid adhesion, and result in their exfoliation from the testis. Carbendazim-induced germ cell loss through MT-based cytoskeleton disrupstion is also known to be stage-specific since early-stage elongating spermatids embedded deep inside the epithelium are less susceptible to carbendazim-induced exfoliation^33^. Subsequent studies have shown that the toxicant-induced retention of elongating/elongated spermatids in the seminiferous epithelium is due to the absence of MT-conferred track-like ultrastructures that help transport spermatids across the epithelium in preparation for their release at spermiation. Failure of the spermatid-specific anchorage device known as apical ES at the Sertoli cell-spermatid interface is thought to underlie this observation^36^.

For the most part, the molecular mechanism(s), in particular those involving signaling protein(s), by which toxicants induce MT disorganization remain unknown. There are reports in the literature that demonstrate that PFOS induces oxidative hepatic damage by triggering an enhanced production of reactive oxygen species (ROS) in rats through the Nrf2 (NF-E2-related factor 2)-dependent mechanism^37^. PFOS also reduces testosterone production by rat fetal Leydig cells following in utero exposure of mother rats to this toxicant^38^, and is capable of inducing apoptosis in mouse Leydig cells^39^. However, the underlying mechanism(s) or involving molecule(s) have yet to be identified. Studies using rat primary Sertoli cell cultures or rat testes have shown that FAK, in particular p-FAK-Tyr^407^, plays a crucial role in regulating Sertoli cell BTB dynamics^22,26,40–42^. For instance, a knockdown of FAK by FAK-specific siRNA duplexes was found to perturb Sertoli cell TJ-permeability barrier function^42^. Subsequent studies have shown that there is an endogenously generated small regulatory RNA called miR-135b produced by mammalian cells that silences FAK^43,44^, and miR-135b was observed to silence FAK in rat Sertoli cells cultured *in vitro* ^26^. Moreover, this miR-135b FAK-specific miRNA was found to worsen the PFOS-induced Sertoli cell TJ-barrier disruption^26^, implicating its likely involvement in PFOS-mediated Sertoli cell injury. Indeed, overexpression of p-FAK-Y407E in rat Sertoli cells was found to block the PFOS-induced disruptive changes in actin microfilament organization^26^. As anticipated, the use of a human p-FAK mutant, p-FAK-Y407E, was shown herein to be effective in rescuing human Sertoli cells from the damaging effects of PFOS on both actin- and MT-based cytoskeletons.

In this context, it is noted that the molecular basis that overexpression of the constitutively active p-FAK-Tyr407 mutant to rescue Sertoli cells from the damaging effects of PFOS on the Sertoli cell cytoskeletal organization remains unknown. However, an earlier report using rat Sertoli cells has shown that overexpression of this p-FAK-Tyr^407^ mutant enhances the kinetics of actin polymerization^22^, illustrating future studies shall focus on the role of p-FAK-Tyr^407^ on the polymerization kinetics of actin and MT polymers, and on altering the bundling activity of actin microfilaments and/or cleavage/depolymerization of actin filaments or MT protofilaments. Furthermore, the ability of the phosphomimetic mutant of FAK-Y407E to rescue the damaging effects of PFOS on the Sertoli cell function may due to the fact that phosphorylation of FAK at Tyr407 can negatively regulate auto-phosphorylation of FAK at Try397^45^ since these two phosphorylation sites are adjacent to each other in the naturally folded FAK polypeptide^45^. Additionally, phosphorylation of FAK at Tyr397 is the hallmark of FAK activation by promoting its intrinsic activity^46^. Thus, overexpression of the phosphomimetic mutant of FAK-Y407 may modulate the intrinsic FAK activity, which in turn induces other cellular changes, and by preventing PFOS-induced hyper-activation of FAK. These possibilities must await additional investigations in future studies. Nonetheless, findings reported herein suggest a potential role for manipulating levels of p-FAK-Tyr^407^ to correct male reproductive dysfunction induced by toxicants such as PFOS.

In our studies reported herein, we elected to use PFOS (Mr 500.126) at a concentration of 20–40 µM so that an obvious phenotype could be detected for study purposes, which represents a PFOS concentration at 10–20 µg/ml medium. Thus, in order to see similar effects in humans, similar serum concentrations would be required. Interestingly, a study that examined the levels of PFOS in human serum samples among a group of Chinese subjects had shown a median level of approximately 0.048 µM in 2002^47^. However, the serum level of PFOS increased by 13-fold from year 1999 to 2002^47^. Moreover, the serum level of PFOS in retired fluorochemical production workers was shown to be ranging from 0.29 µM to 6.98 µM in 1998^10^, which is close to the 20 µM level used in our studies. Given the long half-life of PFOS in serum samples in humans, about 5.4-year^10,11^, a considerable level of PFOS could build up in human subjects in particular among industrial workers or general public who live in contaminated areas. Furthermore, while the production of PFOS has ceased since the late 2000s in the U.S., Canada and Europe, but PFOS continues to be widely used in China and several other countries around the globe in consumer products by serving as a stain repellent and fabric protector^1,9^. Thus, it is expected that the serum level of PFOS is likely to rise among men who live in contaminated areas in particular in industrial workers producing or utilizing PFOS.

In summary, we have demonstrated that human Sertoli cells, similar to rat Sertoli cells, are highly susceptible to the toxic effects of PFOS when cultured *in vitro*. Furthermore, overexpression of a constitutively active mutant of FAK - p-FAK-Y407E - is capable of protecting Sertoli cells from the damaging effects of PFOS. Model systems employing mitotically actively cultured human Sertoli cells should provide a valuable research tool to assess environment toxicants in male reproductive dysfunction.

## Materials and Methods

### Human Sertoli cells

Human Sertoli cells obtained from 4 deceased donors at 15, 23, 36 and 40 years of age (Table 1) were characterized as previously described^15^. These human Sertoli cells were obtained from MandalMed (San Francisco, CA) which also offers these cells for research and purchase through Lonza (Walkersville, MD) to investigators worldwide. Informed consents were obtained from all involving human subjects. These cells were shipped on dry ice and stored in liquid nitrogen upon arrival. The use of Sertoli cells for experiments reported herein was approved by the Rockefeller University Institutional Animal Care and Use Committee (IACUC) with Protocol Numbers 15–780-H. All methods and experimental protocols used in relevant studies and experiments reported herein, including the use of PFOS, primary Sertoli cell cultures, and transfection experiments using recombinant DNA materials including the FAK full-length cDNA and its phosphomimetic (constitutively active) and non-phosphorylatable (constitutively inactive) mutants were carried out in accordance with the relevant guidelines, including any relevant details, and approved by the Rockefeller University Laboratory Safety and Environmental Health, the Rockefeller University Institutional Biosafety Committee (IBC), and the Rockefeller University Comparative Bioscience Center (CBC).

**Table 1:** Growth times of human Sertoli cells cultured *in vitro*.

Donor age (years)	Thawing of cells	Growth medium	Time needed to reach ~70–80% confluence (days)					
			P1	P2	P3	P4	P5	P6
36	Time 0	F12/DMEM + 10% FBS + S/P (1:100)*	21	8	13			
40			10	5	5	7	5	8
15			22	12	10	12		
23			23	19	11			

*FBS, fetal bovine serum; S, streptomycin; P, penicillin; P1-6, cell passage 1-6.

### Human Sertoli cell cultures

Human Sertoli cells were cultured and maintained as earlier described^16^, with minor modifications. Briefly, a vial of cryopreserved human Sertoli cells containing ~1 × 10^6^ cells stored in cryogenic liquid nitrogen tank were thawed in a 37 °C water bath, transferred immediately to an Erlenmeyer flask containing 50-ml Dulbecco’s Modified Eagle’s Medium/Ham’s F12 Nutrient Mixture (DMEM/F12) culture medium (Thermo Fisher Scientific), supplemented with 10% (v/v) fetal bovine serum (FBS, Thermo Fisher Scientific) and penicillin (100 units/ml)/streptomycin (100 mg/ml) (Thermo Fisher Scientific). Thereafter, cells suspended in each 10-ml DMEM/F12 medium were plated onto a 100-mm cellBIND^®^ culture dishes (Corning), to a total of 5 dishes. Human Sertoli cells were cultured in this DMEM/F12 medium at 35 °C in a CO_2_ incubator with 5% CO_2_/95% air (v/v) in a humidified atmosphere. Cells were found to remain mitotically active (Table 1). Medium was replaced every 3–4 days. For subculture, cryopreservation, immunofluorescence microscopy or to monitor the human Sertoli cell TJ-permeability function, cells at 70–80% confluence were washed twice with sterile PBS (10 mM sodium phosphate, containing 0.15 M NaCl, pH 7.4 at 22 °C), and were then re-suspended by treatment with trypsin (0.05%)-EDTA (0.02%) solution (Thermo Fisher Scientific) for ~3 to 5 min. Trypsinization was terminated by FBS-containing F12/DMEM, and cells were collected by centrifugation at 100 *g* for 5 min at room temperature to remove trypsin-containing medium. Cell density was then determined by using a hematocytomer. Cells used for all the experiments reported herein were from the third to the sixth passage (P), and pilot experiments were performed to optimize the culture conditions and to confirm their reproducibility. For immunoblotting (IB), human Sertoli cells were plated on cellBIND^®^ 24-well dishes. For immunofluorescence analysis (IF), cytotoxicity assay and assay to monitor Sertoli cell TJ-barrier function by quantifying TER (transepithelial electrical resistance) across the Sertoli cell epithelium, human Sertoli cells were plated on cover glasses, 96-well culture plates, and bicameral units (Millicell), respectively, which were coated with 2 μg/cm^2^ human fibronectin (BD Biosciences). Human fibronectin was prepared as a 1 mg/ml stock in sterile MilliQ water according to the manufacturer’s instruction and was subsequently diluted in sterile PBS, which was then used to coat the dishes, coverslips or bicameral units without agitation after plating, which were then air-dried at room temperature inside a culture hood, similar to the use of Matrigel as described^48^. For all experiments reported herein, freshly seeded human Sertoli cells on dishes and coverslips were allowed to reach ~70–80% confluency before they were used for IB and IF, respectively, which usually took ~4–5 days. On the day these cells were used for IB or IF, they were counted as cells at time 0.

### Treatment of human Sertoli cells with perfluorooctanesulfonate (PFOS)

PFOS (Mr 500.126) obtained from Sigma-Aldrich was dissolved in DMSO at 100 mM as a working stock solution. Human Sertoli cells at ~80% confluency were serum-starved for 5 hr. Thereafter, cells were then treated with 20 and/or 40 μM PFOS *vs*. the same amount of DMSO alone (vehicle control) for 24 hr. The selected concentrations of PFOS used in our experiments were based on pilot experiments and earlier reported^26,49^.

### Preparation and overexpression of human FAK full-length cDNA (WT, wild type) and its constitutively active and inactive mutants in human Sertoli cells

The pcDNA3/FAK plasmid DNA containing the full-length human FAK cDNA prepared as earlier described^50^ was a gift from Dr. William G. Cance of the Department of Surgical Oncology, Roswell Park Cancer Institute (Buffalo, NY). The human FAK cDNA was subcloned into the *Kpn*I (sense) and *Not*I (anti-sense) sites of mammalian expression vector pcDNA3.1(+) (Thermo Fisher Scientific) designated pcDNA3.1(+)/FAK-WT using primer pair 2 and 3 (Table 2) – i.e., the full-length human FAK cDNA. Two human FAK mutants using this pcDNA3.1(+)FAK-WT to serve as a template were prepared using a three-step mutagenesis PCR approach. For these mutants, one had a point mutation at amino acid Tyr-407 (Y407) to Phe-407 (F407) (i.e., Y407F, non-phosphorylatable and constitutively inactive mutant), and the other one mutated from Tyr-407 (Y407) to Glu-407 (E407) (i.e., Y407E, phosphomimetic and constitutively active mutant). In brief, the constitutively inactive and active mutants were obtained as follows. For constitutively inactive mutant: (i) the first PCR was performed using primer pair 1 and 4 (Table 2) with the human FAK cDNA from pcDNA3.1/FAK-WT plasmid as a template (for 30 cycles); (ii) a second PCR was performed using the PCR product from step (i) as the only primer and pcDNA3.1/FAK-WT plasmid DNA as the template for 1 cycle; and (iii) a third PCR was performed using the PCR mixture from step (ii) with primer pair 2 and 3 to generate the mutant (30 cycles). For constitutively active mutant, it was obtained similar to the constitutively inactive mutant except that primer pair 1 and 5 (Table 2) were used in step (i). The two mutants were designated pcDNA3.1(+)/FAK-Y407F and pcDNA3.1(+)/FAK-Y407E, respectively. All DNA constructs including the WT and mutants (and their corresponding mutation sites) were confirmed by direct nucleotide sequencing at Genewiz (South Plainfield, NJ). For IB and IF, human Sertoli cells at ~80% confluency were transfected with plasmid DNA at 0.5 μg per well using Lipojet^TM^ *In Vitro* Transfection Reagent (SignaGen Laboratories) at a ratio of 2 μl transfection medium:1 μg plasmid DNA in DMEM/F12 supplemented with 1%FBS according to the manufacturer’s instructions. After 24 hr, cells were rinsed with DMEM/F12 medium twice and then cultured in fresh medium for an additional 24 hr. To confirm successful transfection in overexpressing experiments, plasmid DNA was labeled with Cy3 (red fluorescence) using Mirus Label*IT* Tracker Intracellular Nucleic Acid Localization kits.

**Table 2:** Primers used for cloning in this report.

Primer Number	Primer Name	Primer orientation	Primer sequence (5′-3′)
1	pcDNA3.1-T7-S	Sense	GACTCACTATAGGGAGACC
2	hFAK-S	Sense	ACGGTACCATGGACTACCCCTATGATGTGCC
3	hFAK-AS	Anti-sense	ACGCGGCCGCTCAGTGTGGTCTCGTCTGCC
4	hFAK-407-Phe-AS	Anti-sense	CCCTGGTTGAGGGCATGGT***AAA***AGTATCTTCTTCATCTATA
5	hFAK-407-Glu-AS	Anti-sense	CCCTGGTTGAGGGCATGGT***TTC***AGTATCTTCTTCATCTATA

The restriction sites are *Kpn*I (underlined sequence in primer hFAK-S) at the 5′-end and *Not*I (underlined sequence in primer hFAK-AS) at the 3′-end. Mutation sites are annotated in bold/italic. Nucleotide sequences of constructs were confirmed by direct nucleotide sequencing at Genewiz.

### Immunoblot (IB) and immunofluorescence analysis (IF)

To obtain protein lysates, media were removed and human Sertoli cell were treated with lysis buffer (10 mM Tris, pH 7.4 at 22 °C containing 0.15 M NaCl, 1% NP-40 [v/v] and 10% glycerol [v/v], freshly supplemented with protease and phosphatase inhibitors (Sigma) at a 1:100 dilution). Immunoblot data were scanned and quantified by using Scion Image (v4.0.3.2; Scion Corporation; http://scion-image.software.informer.com/). Uncropped images of immunoblots shown in Figs 1B, 3A, 4A and 5B were shown in the corresponding images in Figures S1, S2, S3 and S4. For IF analysis, cells were fixed either in 4% paraformaldehyde (PFA) (w/v) in PBS at room temperature or ice cold methanol for 10 min, to be followed by permeabilization in 0.1% TritonX-100 (v/v) in PBS. F-actin was visualized with either FITC-phalloidin (green fluorescence) or rhodamine-phalloidin (red fluorescence) (Thermo Fisher Scientific). Images were captured using a Nikon Ds-Qi1Mc-U2 camera in a Nikon 90i motorized fluorescence microscope system, and acquired using the NIS-Elements AR software (Version 3.2; Nikon Instruments). Antibodies and conditions used for all experiments in the study were listed in Table 3. Results shown herein for IF analysis were representative data of an experiment which was repeated at least three times using different batches of human Sertoli cells that yielded similar observations. For all IF analysis, negative controls were performed in all immunofluorescence experiments in which the primary antibody was substituted with the corresponding IgG from normal serum (e.g., rabbit or mouse IgG). For instance, negative controls for IF analysis of α-tubulin and EB1 were shown in Figure S5. For IB, data shown were representation data of an experiment from n = 3 independent experiments using human Sertoli cells from different donors. These experiments did not take into account the pilot experiments that were used to establish optimal experimental conditions.

**Table 3:** Antibodies used in this report.

Antibody (RRID)	Host species	Vendor	Catalog no.	Working dilution	
				IB	IF
Actin (AB_630836)	Goat	Santa Cruz Biotechnology	sc-1616	1:300	
Arp3 (AB_476749)	Mouse	Sigma-Aldrich	A5979	1:3000	1:50
α-tubulin (AB_2241226)	Mouse	Abcam	ab7291	1:1000	1:300
β-catenin (AB_138792)	Rabbit	Thermo Fisher Scientific	71–2700	1:250	1:100
β-tubulin (AB_2210370)	Rabbit	Abcam	ab6046	1:1000	
Claudin 11 (AB_2276205)	Rabbit	Abcam	ab53041	1:500	
EB1 (AB_397891)	Mouse	BD Biosciences	610534		1:200
EB1 (AB_2141629)	Rabbit	Santa Cruz Biotechnology	sc-15347	1:200	
Eps8 (AB_397544)	Mouse	BD Biosciences	610143	1:5000	1:50
N-cadherin (AB_2313779)	Mouse	Thermo Fisher Scientific	33–3900	1:200	1:100
Goat IgG-HRP (AB_634811)	Bovine	Santa Cruz Biotechnology	sc-2350	1:3000	
Rabbit IgG-HRP(AB_634837)	Bovine	Santa Cruz Biotechnology	sc-2370	1:3000	
Mouse IgG-HRP (AB_634824)	Bovine	Santa Cruz Biotechnology	sc-2371	1:3000	
Rabbit IgG-Alexa Fluor 555 (AB_2535850)	Goat	Thermo Fisher Scientific	A-21429		1:100
Rabbit IgG-Alexa Fluor 488 (AB_2576217)	Goat	Thermo Fisher Scientific	A-11034		1:100
Mouse IgG-Alexa Fluor 488 (AB_2534088)	Goat	Thermo Fisher Scientific	A-11029		1:100
Mouse IgG-Alexa Fluor 555 (AB_2535844)	Goat	Thermo Fisher Scientific	A-21422		1:100

Abcam, Cambridge, MA; Santa Cruz Biotechnology, Santa Cruz, CA; Sigma-Aldrich, St. Louis, MO; Thermo Fisher Scientific, Carlsbad, CA; BD Biosciences, San Jose, CA. RRID, Research Resource Identifier.

### Monitoring the human Sertoli cell TJ-permeability barrier function *in vitro*

Human Sertoli cells were seeded on human fibronectin (2 μg/cm^2^)-coated bicameral units (surface area, ~0.6 cm^2^) at approximately 3 ×10^5^ cells/unit. Bicameral units were then placed in 24-well plates with 0.5 ml growth medium each in the apical and basal compartment (see Fig. 1A). Human Sertoli cells were allowed to rest in the bicameral units for two days before quantifying the TJ-permeability barrier by recording the TER across the cell epithelium using the Millipore Millicell-ERS-2 as earlier described^48^ on a daily basis. The day when the first TER measurement was taken was arbitrarily set as day 1. To assess the TJ-permeability barrier, both the treatment and the vehicle control group contained triplicate or quadruplicate bicameral units, and TER across the cell epithelium was recorded. In brief, a set of Millicel ERS Ag/AgCl electrode was placed vertically into each well with the longer end inserted into the basal compartment and the shorter end placed in the apical compartment of the bicameral unit (Fig. 1A). A short pulse of current (20 µA) for ~2 sec was sent through the cell epithelium and the resistance (in ohm) was recorded, which thus quantified the TJ-permeability barrier as described^51^. The electrode was placed on the two opposite ends of a bicameral unit, and these 4 TER readings were averaged as earlier described^49^. Blanks were obtained from fibronectin-coated bicameral units containing equal amount of culture medium without human Sertoli cells. TER experiments were repeated at least 3 times using different batches of Sertoli cells and yielded similar results.

### Cytotoxicity assay and apoptosis assay

Cell proliferation kit II (XTT, sodium 3′-[1-(phenylaminocarbonyl)-3,4-tetrazolium]-bis (4-methoxy-6-nitro) benzene sulfonic acid hydrate) (Roche) was used for quantification of cytotoxicity as described^16^. *In Situ* cell death detection kit (Roche), a TUNEL-based assay, was used to further access the cytotoxicity of PFOS on human Sertoli cells. In short, cells treated with DMSO (vehicle control) *vs*. 10, 20, 40, 80, 100 μM of PFOS for 24 hr were fixed in 4% PFA (w/v) in PBS at room temperature for 1 hr. These cells were then permeabilized in 0.1% TritonX-100 (v/v) in PBS containing 0.1% sodium citrate (w/v) for 2 min on ice and were then incubated with TUNEL reaction mixture for 1 hr at 37 °C in complete darkness. Nuclei of apoptotic cells were labeled with green fluorescence.

### Statistical analysis

All experiments were repeated using human Sertoli cells from at least three different donors and summarized in Table 1. Each data point was expressed as a mean ± SD of *n* = 3 independent experiments or quadruple bicameral units (to quantify TJ-permeability barrier function) within one experiment. Statistical differences between sample groups were determined by Student’s *t-*test (for paired comparisons), or one-way analysis of variance (ANOVA) followed by Dunnett’s test (for multiple-group comparisons) using the GB-STAT (Version 7.0) statistical analysis software package (Dynamic Microsystems, Silver Spring, MD).

## Electronic supplementary material

Supplementary Information
