# Supplementary material for: Rescue of PFOS-induced human Sertoli cell injury by overexpressing a p-FAK-Y407E phosphomimetic mutant
Source: Sci Rep. 2017 Nov 17;7:15810. doi: 10.1038/s41598-017-15671-4 (PMC5693862; doi:10.1038/s41598-017-15671-4)

Supplementary Information

Chen HQ et al. Rescue of PFOS-induced human Sertoli cell injury by overexpressing a p-FAK-Y407E phosphomimetic mutant

Figure S1. Immunoblots that illustrate the effects of PFOS on the expression of selected BTB-associated proteins. These are uncropped blot images corresponding to data shown in Figure 1B.

Figure S2. Immunoblots that illustrate the effects of PFOS on the expression of selected actin regulatory and signaling proteins. These are uncropped blot images corresponding to data shown in Figure 3A.

Figure S3. Immunoblots that illustrate the effects of PFOS on the expression of selected MT regulatory protein EB1. These are uncropped blot images corresponding to data shown in Figure 4A.

Figure S4. Immunoblots that illustrate an increase in the steady-state of FAK protein level following overexpression of different FAK constructs including FAK mutants. These are uncropped blot images corresponding to data shown in Figure 5B.

Figure S5. Immunofluorescence analysis of α-tubulin and EB1 in Sertoli cells. Immunofluorescence microscopy was used to examine the organization of microtubules (MTs) and EB1 (a MT stabilizing protein) in Sertoli cells using an anti-α-tubulin antibody (note: α-tubulin is a building block of MTs) and an anti-EB1 antibody (see Table 3). The corresponding negative control was shown using either normal mouse or normal rabbit IgG. This thus confirms the staining of α-tubulin and EB1 in Sertoli cells. It is noted that these antibodies have earlier been characterized in our laboratory [1,2]. These findings also support data shown in Figures 4 and 6 in the main text in which the staining for α-tubulin and EB1 are specific for the corresponding proteins.

References:

1 **Tang, E. I., Mok, K. W., Lee, W. M. & Cheng, C. Y. EB1 regulates tubulin and actin cytoskeletal networks at the Sertoli cell blood-testis barrier in male rats - an in vitro study. *Endocrinology* 156, 680-693 (2015).**

2 **Tang, E. I., Lee, W. M. & Cheng, C. Y. Coordination of actin- and microtubule-based cytoskeletons supports transport of spermatids and residual bodies/phagosomes during spermatogenesis in the rat testis. *Endocrinology* 157, 1644-1659, doi:10.1210/en.2015-1962 (2016).**

Figure S1 (Chen et al.)

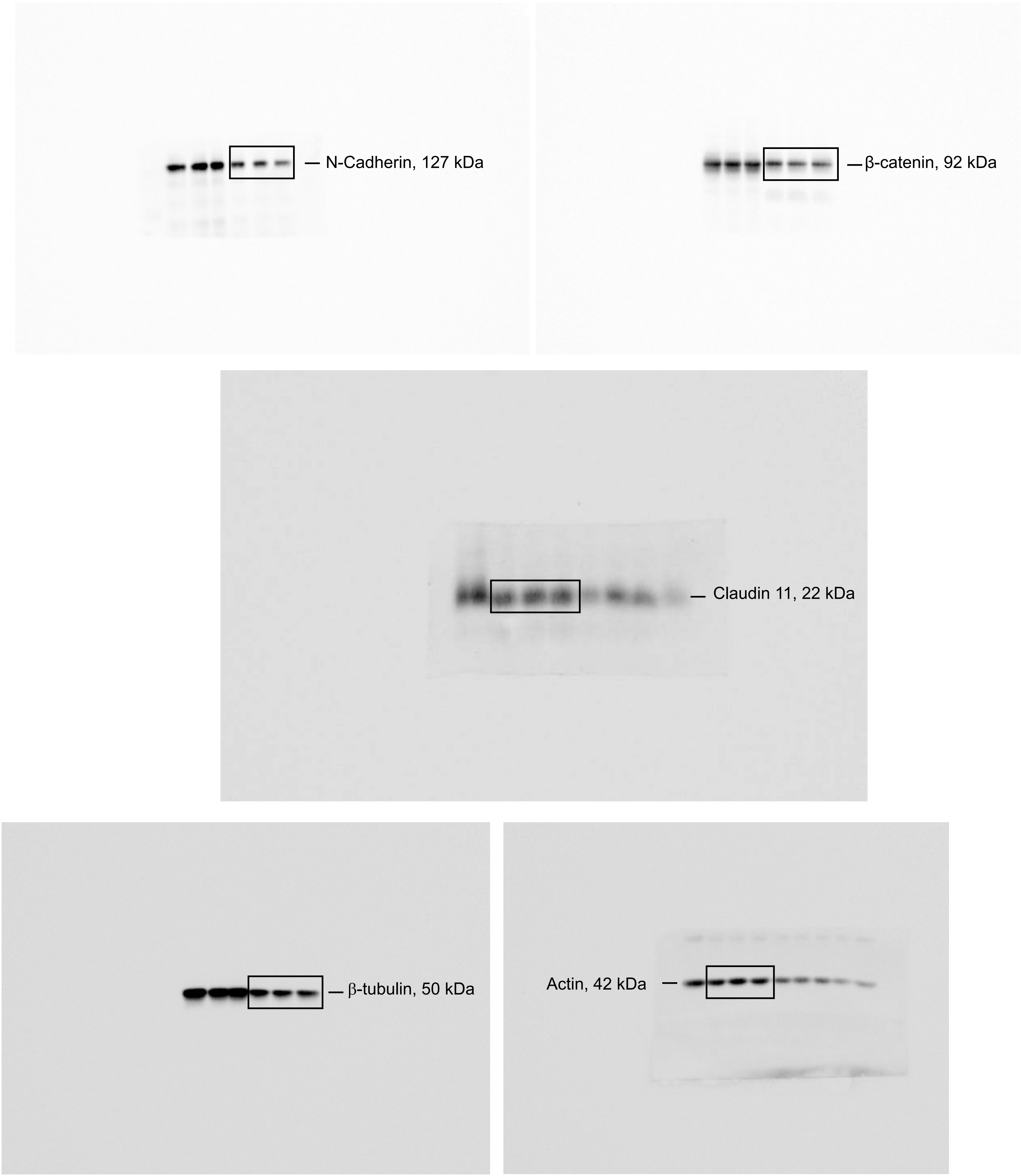

Figure S2 (Chen et al.)

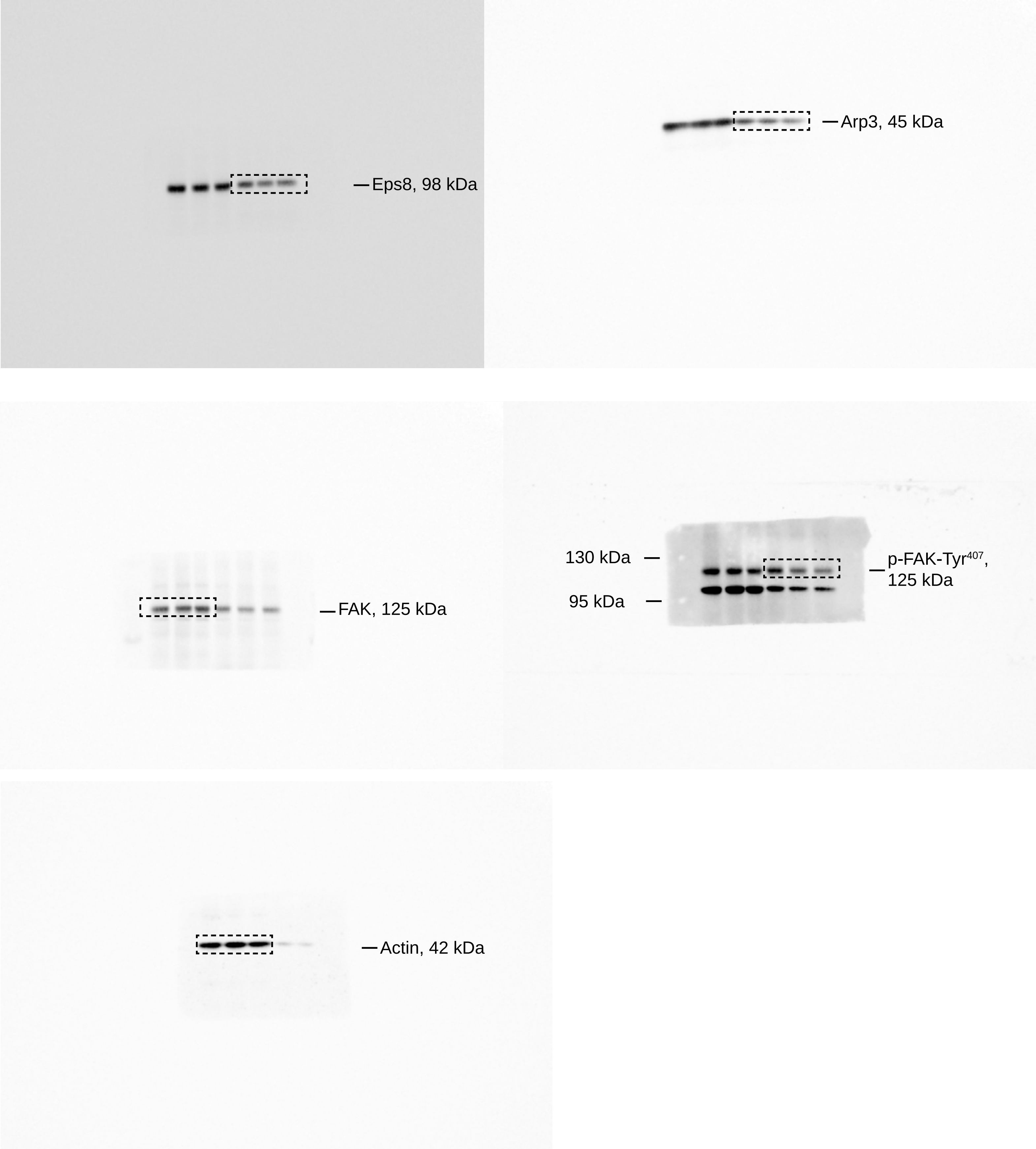

Figure S3 (Chen et al.)

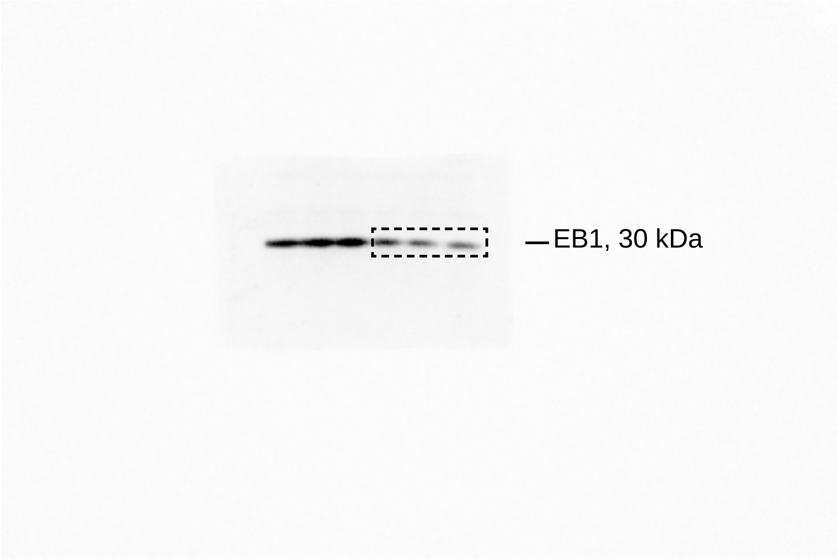

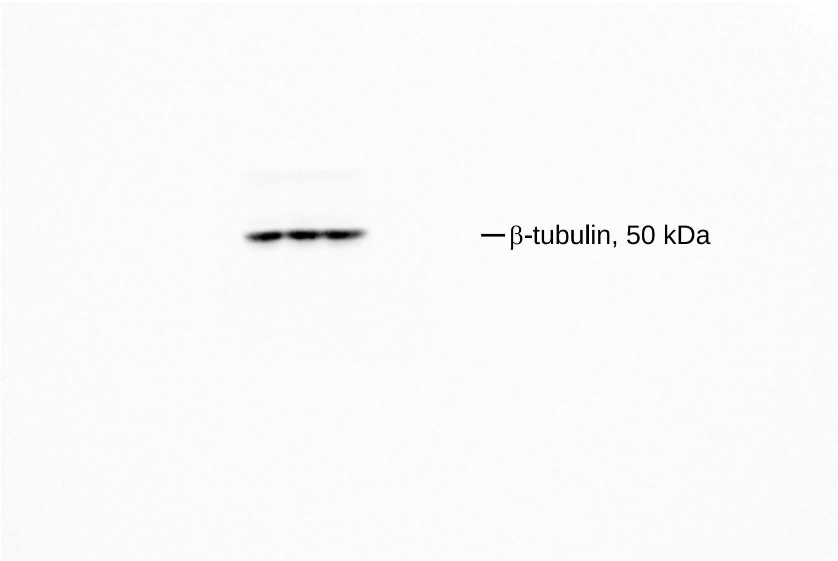

Figure S4 (Chen et al.)

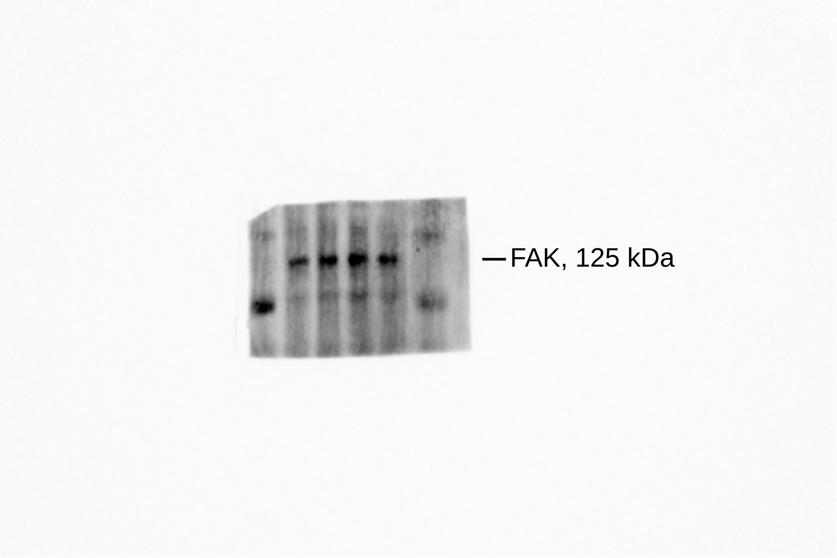

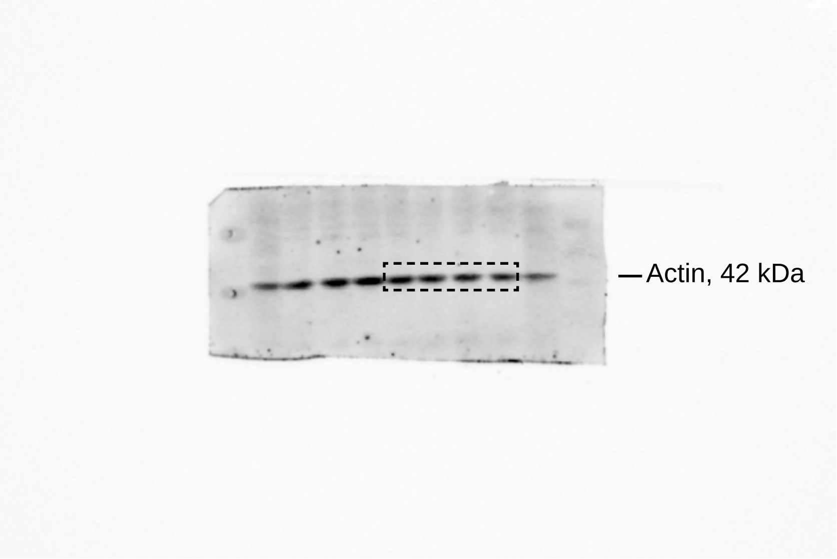

Figure S5 (Chen et al.)

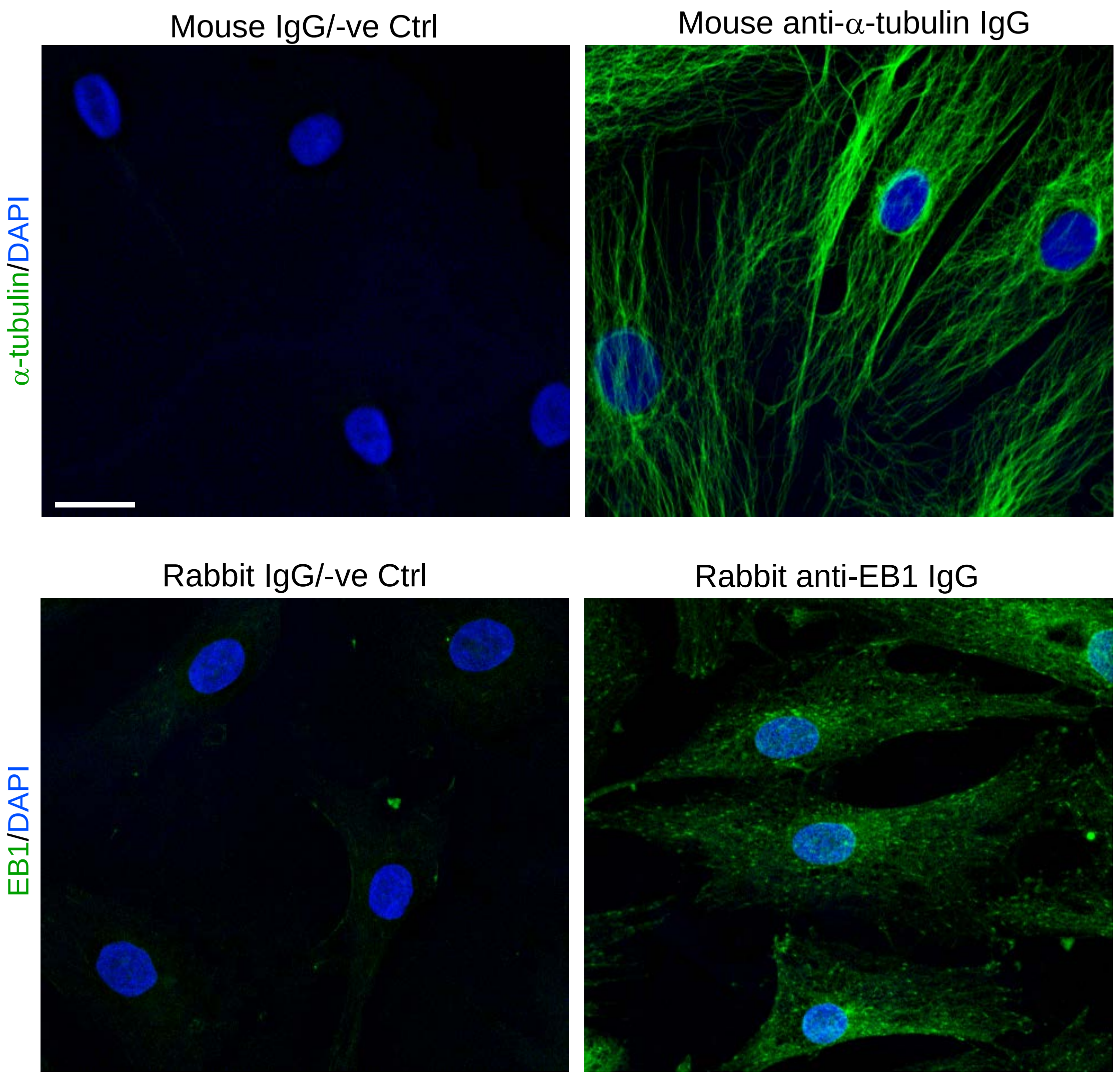

Supplement: Supplementary file 1 — Supplementary Information [file 41598_2017_15671_MOESM1_ESM.pdf]
